# Changes of Locoregional Skin Temperature in Neonates Undergoing Laser Needle Acupuncture at the Acupuncture Point Large Intestine 4

**DOI:** 10.1155/2015/571857

**Published:** 2015-04-02

**Authors:** Stefan Kurath-Koller, Gerhard Litscher, Anna Gross, Thomas Freidl, Martin Koestenberger, Berndt Urlesberger, Wolfgang Raith

**Affiliations:** ^1^Division of Neonatology, Department of Paediatrics and Adolescent Medicine, Medical University of Graz, 8036 Graz, Austria; ^2^Research Unit for Complementary and Integrative Laser Medicine, Research Unit of Biomedical Engineering in Anesthesia and Intensive Care Medicine and TCM Research Center Graz, Medical University of Graz, 8036 Graz, Austria; ^3^Division of Pediatric Cardiology, Department of Paediatrics and Adolescent Medicine, Medical University of Graz, 8036 Graz, Austria; ^4^Research Group for Paediatric Traditional Chinese Medicine, TCM Research Center Graz, Medical University of Graz, 8036 Graz, Austria

## Abstract

Laser acupuncture bears a potential risk for the skin surface, especially in neonates whose skin has histological and physiological peculiarities. We evaluated thermal changes of skin temperature in neonates during laser acupuncture by using a thermal camera (Flir i5, Flir Systems Inc., Portland, USA). Laserneedles (Laserneedle GmbH, Glienicke/Nordbahn, Germany) were fixed to the skin at Large Intestine 4 (LI 4, *Hegu*), bilaterally. Before application of laser acupuncture (685 nm, 15 mW, 500 *μ*m), as well as after 1, 5, and 10 min, thermographic pictures of both hands were taken. The measuring was carried out on the 23rd day after birth (20 neonates, mean postmenstrual gestational age 38 + 2, mean weight 2604 g). Compared to the initial temperature of 34.2°C on the right hand, the skin temperature had increased to 35.3°C (*P* < 0.05) after 5 min and up to 36.1°C (*P* < 0.05) after 10 min of stimulation. Equally, on the left hand, an increase of the skin temperature from 34.5°C to 35.9°C (*P* < 0.05) and 35.9°C (*P* < 0.05) was measured. The highest measured skin temperature after 10 min of stimulation amounted to 38.7°C, without any clinically visible changes on the skin surface.

## 1. Introduction

Traditional Chinese medicine (TCM) is reported as one of the most popular treatments for children [1]. TCM includes (i) massage therapy (*Tuina*), (ii) moxibustion, and (iii) different kinds of acupuncture and acupressure [2]. The most common additional applications for acupuncture in the pediatric population are from the area of pediatric oncology [3–5] suggesting that acupuncture is an appropriate adjunctive treatment for chemotherapy-induced nausea/vomiting [6] and for different kinds of pain [7–9]. In addition, both a recent and a 3-year-old systematic review of acupuncture for children and newborns found that acupuncture is a safe treatment when performed by trained and licensed acupuncturists [10, 11]. Both therapy and prevention of pain [12] are warranted to avoid acute physiologic reactions [13] and there are data that pain-related stress can induce long-term effects, like poorer cognition and motor functions, as well as lead to altered cortisol levels in patients at school age [14, 15]. The limited data available suggests that needle acupuncture is a safe nonpharmacologic treatment option for the reduction of pain [16, 17] and agitation in term and preterm infants [11, 18, 19] and in the therapy of infantile colic [20–24]. But at the moment it is unknown whether repeated needle stimulation may alter sensory processing and responses to subsequent painful stimuli, in the same manner like heel sticks used on infants [13, 14]. Therefore, most acupuncturists who treat children use special techniques, including nonneedle methods, (e.g., lasers and vigorous massage or tapping) to stimulate points along the energy meridians. The development of laser acupuncture [25] has opened a new door in the treatment of children and is continuing to do so. The result is a noninvasive therapeutic approach which, additionally, rules out any existing risks of infection [11, 25, 26]. At the moment there are a few trials [27–30] and a few case series promising positive results in laser acupuncture treatment for children [31–33] and in neonates with neonatal abstinence syndrome (NAS) [34, 35]. Furthermore,* in vitro* experiments demonstrated that irradiation with red laser light of 657 nm stimulates adenosine triphosphate (ATP) release [36] which is associated with an intracellular free Ca2+ rise in human mast cells, similar to needle acupuncture [25]. However laser acupuncture has to be used with caution bearing a potential risk for eyes and skin surface. The neonatal skin is thinner and represents a much more sensitive barrier than the skin of adults [37], putting it at risk for potential thermal damage. There has, however, only been very little literature about laser acupuncture in neonates dealing with peripheral and central changes [38, 39] and clear recommendations are still missing [40–42]. Therefore, the aim of this study was to evaluate thermal changes of skin temperature in neonates by the use of an infrared thermal camera to get more data about the safety for the use of laser acupuncture in this vulnerable population.

## 2. Materials and Methods

### 2.1. Probands

The probands were former premature infants hospitalized at the Division of Neonatology, Department of Paediatrics and Adolescent Medicine at the Medical University Graz who were all tested in the sleep lab before being discharged from hospital. Their parents were informed about the examination and gave their prior written consent. The study itself was submitted to the Ethics Committee of the Medical University of Graz and approved.

All together 20 neonates (12 male, 8 female, mean gestational age (GA) [43] 35 + 0 weeks of pregnancy, and mean birth weight 2261.2 g) were included in the study. On average, the measuring was carried out on the 23rd day after birth (chronological age) with a mean postmenstrual GA 38 + 2 weeks of pregnancy [43] and a mean weight of 2604 g at the time of examination.

Table 1 gives data of the children involved in the study.

### 2.2. Laser Acupuncture

The laserneedle used for acupuncture (Laserneedle GmbH, Glienicke/Nordbahn, Germany) provides continuous laser light with a wavelength of 685 nm and an output power of 15 mW per laserneedle. The diameter of the laserneedle is 500 *μ*m.

### 2.3. Procedure

**Figure 1:**
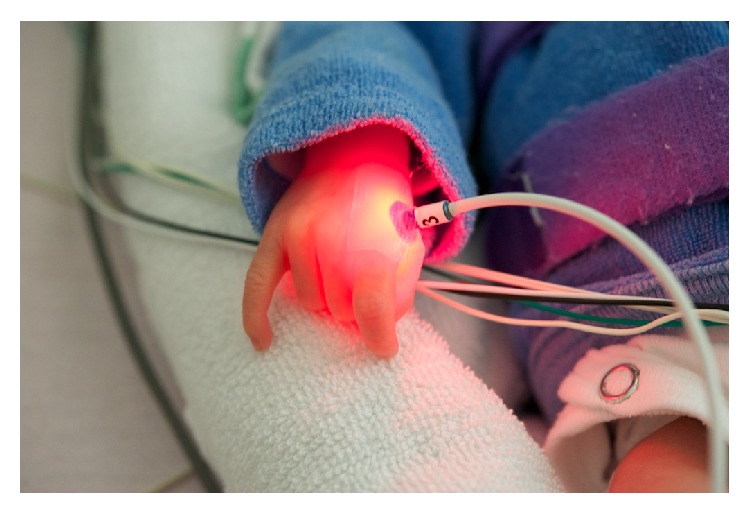
Demonstration of the applied laserneedle at the acupuncture point Large Intestine 4 (LI 4,* Hegu*).

The probands were comfortably placed in a Babytherm 8000 incubator (Dräger GmbH, Lübeck, Germany) in the course of the sleep lab examination. In all incidents, a time period of 10 min of waiting was respected before applying the laserneedles to give the skin of the neonates a chance to acclimatize to the temperature. Before laser acupuncture was performed, the skin at the acupuncture point was disinfected and the laserneedles were fixed to the skin with a special adhesive tape, bilaterally at Large Intestine 4 (LI 4,* Hegu*) (Figure 1).

Then, after a waiting period of 25 minutes, after applying the laserneedles, laserneedle acupuncture was performed simultaneously on both arms at the LI 4 (*Hegu*) point. The first stimulation lasted for 5 min. After an interval of 10 minutes, a second stimulation was carried out in the same way but this time lasting for 10 minutes.

### 2.4. Acupuncture Point

*Large Intestine 4 (LI4, Hegu).* LI4 (*Hegu*) is located on the large intestine meridian, on the dorsum of the hand, between the first and second metacarpal bone on the radial side [42].

### 2.5. Thermography

Before application of laser acupuncture, as well as after 1 min, 5 min, and 10 min, respectively, thermographic pictures of both the left and right hands were taken by means of a thermal camera (Flir i5, Flir Systems Inc., Portland, USA). Subsequently, the warmest spot was identified and reidentified and compared throughout the course of time.

**Figure 2:**
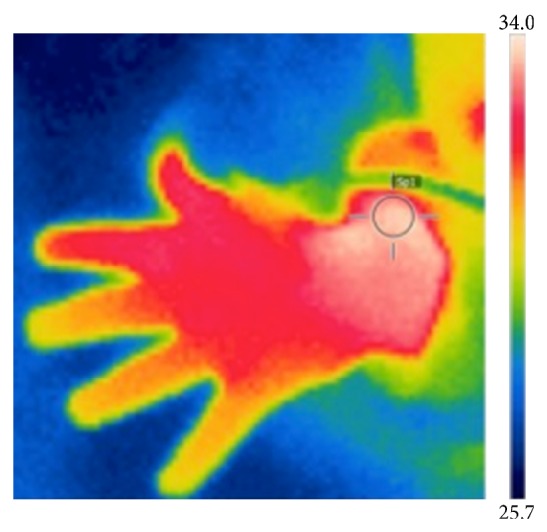
An example of the pictures taken with the use of the thermal camera after laserneedle stimulation. Values are presented as °C.

Figure 2 gives an example of the pictures taken with the thermal camera.

Additionally in the course of the examination, any parameters measurable in the context of polygraphy, such as the heart rate, oxygen saturation, end-expiratory CO_2_, and breathing movements including electroencephalography, were recorded and analyzed. Throughout the examination, the ambient temperature and humidity were kept constant. At that point, in time of the examination, no medication modifying the blood circulation was administered.

### 2.6. Statistics

All data were taken as a mean value ± SD (standard deviation). The statistical evaluation was done using an ANOVA test (Analysis of Variance) for repeated measuring and the Tukey test, respectively (SigmaPlot 12.0, Systat Software, Chicago, USA). The level of significance was defined as *P* < 0.05.

### 2.7. Safety Precautions

The physician carrying out the acupuncture is a general physician and a specialist in pediatric and adolescent medicine as well as in neonatology and intensive care medicine; he also has a diploma in acupuncture and more than 10 years of experience.

The acupuncturist and any other person in the room (e.g., the nurse and the parents) put on specific protective glasses to avoid retinal damage. However, for newborn infants there are no adequate protective glasses available. Therefore the eyes were covered using an eye protector (Natus Biliband Eye Protector, Natus Med. Inc., San Carlos, USA) to avoid any injury to their eyes, as previously described [38, 44].

## 3. Results

Altogether, 360 thermographic measurements were taken of the above-mentioned measuring points (9 on the right hand and 9 on the left hand of each infant). 

*Findings on the Right Hand*. Compared to the initial temperature of 34.2°C, the skin temperature had significantly increased to 35.3°C (*P* < 0.05) after 5 min of stimulation. Equally, a significant rise in temperature was measured again after 10 min of stimulation (36.1°C) (*P* < 0.05). The maximum measured skin temperature after stimulation was found to be 38.3°C.

*Findings on the Left Hand*. Compared to the initial temperature of 34.5°C, the skin temperature had significantly increased to 35.9°C (*P* < 0.05) after 5 min of stimulation. Equally, a significant rise in temperature was measured again after 10 min of stimulation (35.9°C) (*P* < 0.05). The maximum measured skin temperature after stimulation was found to be 38.7°C. Maximum temperatures on the right and left hand were both measured for the same patient.

There was no mention of patient distress or discomfort during laser acupuncture.

The parameters simultaneously measured during the examination (heart rate, oxygen saturation, and end-expiratory CO_2_ (CO_2_, carbon dioxide)) and breathing movements including electroencephalography showed no significant changes.

**Figure 3:**
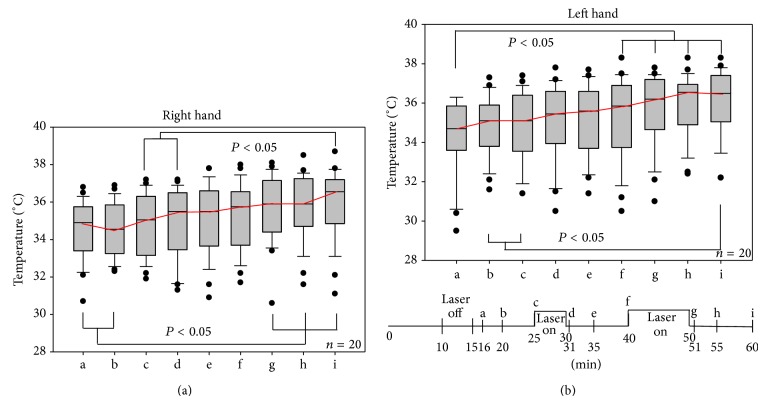
Results and time curve of the examination of the left and the right hand.

Figures 3(a) and 3(b) display the data of temperature measurements of the right and the left hand.

## 4. Discussion

Thermography, that is, the temperature measured by means of an infrared camera, represents a measuring procedure already used for acupunctural research in adults [45, 46]. Thermography is well established for children [47] and neonates [48, 49], because with this method the visualization of temperature distributions of a high local resolution is possible. Furthermore, the diagnostic procedure does not have any radiation-like side effects, is quick to perform, can be repeated as frequently as necessary, and is a noncontact form of examination.

Raith et al. [39] showed that the use of laser acupuncture applied at 10 mW over a period of 10 minutes, repetitively, does not cause dangerous thermal effects on the neonatal skin. However, the acupuncture effect of laser stimulation depends on the power density at the acupuncture point. Litscher and Schikora [50] hypothesized that a higher energy dosage caused by either higher laser output power or a longer radiation time might result in a more significant increase in skin temperature. Now, comparing our results using the 15 mW laserneedle stimulation over 5 and 10 minutes, constituting the same radiation time as used in the prior study of Raith et al. [39], and using the 10 mW laserneedle stimulation, we found the following: a stepwise statistically significant increase in skin temperature either between placebo (the laserneedle fixed at the LI4 acupuncture point without stimulation) and 5-minute laser stimulation or between 5-minute and 10-minute laser stimulation was found in both study protocols, using 10 mW and 15 mW laserneedle stimulation. However, there was an increase in peak skin temperature of 0.8°C comparing our 15 mW laser stimulation to the prior performed 10 mW laser stimulation performed by Raith et al. [39] (37.9°C using 10 mW versus 38.7°C using 15 mW). In both the 10 mW and the 15 mW laserneedle stimulation, the peak temperature was measured after the 10-minute stimulation course. However, the warming of the skin after 5 and 10 min, respectively, of laserneedle acupuncture using a laserneedle (15 mW/685 nm) does not reach such high skin temperature compared to the local temperatures reached in transcutaneous blood gas measuring, as widely used in certain neonatal intensive care units all over Germany, Switzerland, and Austria [51, 52].

Regarding energy dosage we calculated that laser acupuncture applied at 15 mW over a period of 5 minutes, using a laserneedle with a diameter of 500 *μ*m, results in an energy dosage of about 2.3 kJ/cm² and over 10 minutes in an energy dosage of about 4.6 kJ/cm².

We chose LI 4 due to its easy accessibility and because it can be quickly and easily found, being located at the dorsum of the hand. Since examination took place throughout the polysomnography and laserneedles had to be applied, accessibility played an important part in choosing the acupuncture point. Moreover we attempted to select an acupuncture point that is one of the most commonly used acupuncture points especially for newborn infants and children; LI 4 is the most used acupuncture point for the therapy of infantile colic, and current evidence suggests that it is effective and safe [11, 20, 22, 23]. Further evidence for efficacy and safety comes from observational studies with 913 infants [24] and a case report in an infant suffering from NAS where laser acupuncture at LI 4 was found to positively affect feeding and improve calmness [35].

It has been well established that acupuncture stimulation at LI 4 elicits local microcirculation [53, 54] as an indicator of a reduced sympathetic activity [54, 55]. However, the question as to whether or not changes in the surface temperature in connection with the application of laser acupuncture in neonates are to be understood as changes in the sympathetic activity, and therefore as an effect directly resulting from acupuncture, could not be answered by this study.

## 5. Conclusion

In neonates and infants whose skin is altogether thin and shows physiological and histological peculiarities, it is important to know that there is a potential danger of damage to the skin by using laser acupuncture. In the present study we did not observe any adverse events and the highest measured skin temperature after 10 min of stimulation amounted to 38.7°C. No newborn infant developed clinically visible changes on the skin surface due to laser acupuncture, suggesting that laser acupuncture is safe. However the warming of the skin during/after the use of laser acupuncture in neonates and infants should be treated with caution.

Currently acupuncture should be limited to thoroughly monitored clinical trials, and further studies evaluating short- and long-term effects are urgently needed.

## Figures and Tables

**Table 1:** Demographic data of the 20 participants of the study. Values are mean and standard deviation for continuous data and absolute counts for categorical data.

Investigated neonates	*N* = 20
(Male/female)	(12/8)
Mean GA^∗^	35 + 0
Mean birth weight	2261.2 g (SD = 909.2)
Mean arterial PH from umbilical cord sampling	7.28 (SD = 0.09)
Mean APGAR 1	7.5 (SD = 1.9)
Mean APGAR 5	8.5 (SD = 1.39)
Mean APGAR 10	8.9 (SD = 1.08)

Mean postmenstrual GA^∗^, at the time of investigation	38 + 2
Mean chronological age, at the time of investigation	23 (SD = 15)
Mean weight, at the time of investigation	2604 g (SD = 611.7)

^∗^GA: gestational age in completed weeks [43].

## Abbreviations

TCM: Traditional Chinese medicine
ATP: Adenosin triphosphat
LI 4 (*Hegu*): Large Intestine 4
GA: Gestational age
SD: Standard deviation of the mean
APGAR: Apgar-score
NAS: Neonatal abstinence syndrome.
